# Effectiveness of a Gamification-Based Intervention for Learning a Structured Handover System Among Undergraduate Nursing Students: A Quasi-Experimental Study

**DOI:** 10.3390/nursrep15090322

**Published:** 2025-09-04

**Authors:** Mauro Parozzi, Irene Meraviglia, Paolo Ferrara, Sara Morales Palomares, Stefano Mancin, Marco Sguanci, Diego Lopane, Anne Destrebecq, Maura Lusignani, Elisabetta Mezzalira, Antonio Bonacaro, Stefano Terzoni

**Affiliations:** 1 Department of Biomedicine and Prevention, University of Rome “Tor Vergata”, Via Montpellier 1, 00133 Rome, Italy; 2 Internal Medicine Unit, Rho Hospital, ASST Rhodense, 20017 Rho, Italy; 3 Bachelor School of Nursing, ASST Santi Paolo e Carlo—Presidio San Paolo, Via Ovada 26, 20142 Milan, Italy; 4 Department of Pharmacy, Health and Nutritional Sciences (DFSSN), University of Calabria, 87036 Rende, Italy; 5 Dietetics and Clinical Nutrition Unit, Cancer Center, IRCCS Humanitas Research Hospital, Via Manzoni 56, 20089 Rozzano, Italy; 6 Research Unit Nursing Science, Department of Medicine and Surgery, Campus Bio-Medico di Roma University, 00128 Rome, Italy; 7 Bachelor School of Nursing, IRCCS Humanitas Research Hospital, Via Manzoni 56, 20089 Milan, Italy; 8 Department of Biomedical Science for Health, University of Milan, Via Pascal 32, 20133 Milan, Italy; 9 Department of Women’s and Children’s Health, University of Padova, 35128 Padova, Italy; 10 Department of Medicine and Surgery, University of Parma, Via Gramsci 14, 43125 Parma, Italy

**Keywords:** handover, gamification, serious game, SBAR, nursing education, mental health

## Abstract

**Background/Objectives**: Effective clinical handover is a critical component of nursing care, particularly in mental health settings, where the transfer of clinical and behavioral information is essential for both patients’ and health personnel’s safety. Gamification has emerged as a promising strategy to enhance clinical education, yet few interventions have focused specifically on mental health care contexts. This study aimed to evaluate the effectiveness of a serious game designed to teach the SBAR (Situation, Background, Assessment, Recommendation) handover framework to undergraduate nursing students through a psychiatric care unit scenario. **Methods**: A quasi-experimental pre–post design was employed with a convenience sample of 48 nursing students from a Northern Italian university. Participants completed a test assessing their ability to organize clinical information according to the SBAR model before and after the game intervention. Students’ experience was assessed using the Player Experience Inventory. **Results**: A statistically significant improvement in SBAR application was observed post-intervention. The majority of students reported a positive experience across PXI domains such as Meaning, Challenge, Progress Feedback, and Enjoyment. Comparisons with a previously validated video-based nursing serious game showed a consistent overall pattern in response trends. **Conclusions**: The SG was an effective and engaging educational tool for improving structured handover skills in nursing students. Gamification may represent a valuable complement to traditional instruction in nursing education, especially in high-communication clinical areas such as mental health. Further research is needed to assess long-term retention and to explore more immersive formats.

## 1. Introduction

The handover process is a critical component of nursing care, as it ensures continuity and significantly contributes to patient safety [1]. Extensive research has highlighted that communication failures within health care settings are among the leading causes of adverse events [2,3]. The Joint Commission further identified inadequate handovers as a contributing factor in more than 50% of so-called “never events” reported between 2012 and 2016, including treatment delays, wrong-site surgeries, patient falls, and medication errors [4,5,6]. To enhance the quality of handovers, numerous authors have emphasized the need for structured tools, such as the SBAR (Situation, Background, Assessment, Recommendation) and for specific training on this practice, especially in specialized contexts such as mental health context [7,8], pediatrics [9] or emergencies [10]. Despite this, recent evidence indicates that 62.6% of nursing students have not received specific education on handovers [11] and that 92% of nurses report having acquired handover skills informally, either by observing experienced colleagues or through verbal instructions from clinical tutors [12].

A recent systematic review [13] examined educational interventions aimed at teaching handover methods to nursing students. However, the clinical settings in which these interventions were implemented were limited. Most studies focused on internal medicine, surgical, or laboratory environments, while no training initiatives specific to mental health care were identified. Yet, in this particular setting, the use of structured communication frameworks has been shown to improve the clarity and quality of clinical information transfer, thereby positively affecting handover effectiveness, care quality, staff satisfaction, and the safety of both patients and health care professionals [14,15]. Unlike other clinical contexts, handovers in mental health settings extend beyond the transmission of objective data. They require the ability to recognize emotionally and behaviorally relevant signs, psychiatric instability, and risk factors associated with dysfunctional behaviors, aggression, or disruptions in relational and emotional functioning [16]. Attending to subtle communicative or behavioral cues (such as changes in facial expression, posture, speech patterns, or interaction styles) can be crucial for identifying suicidal ideation, unspoken psychotic symptoms (e.g., hallucinations or delusional thinking), or silent deterioration in a patient’s mental state [16]. These elements are particularly challenging to convey in a structured format, yet their timely and accurate transmission is critical for effective risk assessment and prompt clinical decision-making. In this light, incomplete, disorganized, or ambiguous communication during handovers may significantly impair the adequacy of care and lead to serious clinical consequences, arguably even more so than in other health care environments [15,17,18]. Given these challenges, mental health settings constitute high-intensity educational environments that facilitate not only the integration of theoretical knowledge and communication skills but also the development of clinical sensitivity, expressive precision, and the ability to prioritize and articulate information relevant to patient safety. These contexts are, therefore, especially suitable for training in the use of professional language and structured communication models, such as the SBAR framework, as supported by the current literature [13]. Although several innovative teaching strategies have been adopted in mental health education [19,20,21], including practical workshops, virtual patient scenarios, simulated electronic health records, videos, scenario-based simulations [13] and other gamification approach [22,23], a recent scoping review on gamification strategies in mental health contexts [24] found no evidence of serious games (SGs) specifically designed to teach nursing handover skills in this setting. In light of the lack of mental health-focused SBAR training tools, this study aims to design and evaluate the effectiveness of an educational intervention based on an SG set in a mental health care scenario. The primary objective is to promote the acquisition of structured communication skills in nursing handovers among students by assessing their accuracy in applying the SBAR model for classifying clinical information. As highlighted in other contexts [25,26], gamification might have the potential to enhance SBAR competencies also in mental health settings by fostering motivation [19,27], attention [20,21], and communicative skills [19,22,28] while providing immediate feedback and opportunities for practice [22,28]. These mechanisms align with Kolb’s Experiential Learning Theory [29], Deci and Ryan’s Self-Determination Theory [30,31], and Sweller’s Cognitive Load Theory [32], which together emphasize experiential learning, intrinsic motivation, and optimized cognitive processing in skill acquisition.

## 2. Materials and Methods

A quasi-experimental pre–post study design was employed to assess the effectiveness of a gamified educational intervention aimed at teaching a structured handover framework to undergraduate nursing students enrolled in a university program in Northern Italy. This study was conducted over two consecutive days between November and December 2024. The SBAR framework was selected for two primary reasons: it is the most extensively studied and commonly adopted method in the literature on clinical handovers [9]. Additionally, it has been formally endorsed in various health care settings by the Lombardy Region, where this study took place [33].

### 2.1. Theoretical Framework

The educational intervention was structured on an integrated theoretical framework that included classic theories such as Kolb’s Experiential Learning Theory [29], Deci and Ryan’s Self-Determination Theory (SDT) [30,31], and Sweller’s Cognitive Load Theory (CLT) [33]. Kolb’s theory is one of the most widespread educational frameworks, and it focuses learning development through an experiential circle with reflection, conceptualization, and experimentation processes, building practical and communication skills in clinical settings. At the same time, SDT highlights how satisfying the psychological needs of autonomy, competence, and relatedness is crucial to sustaining students’ intrinsic motivation and engagement during the training intervention. Finally, CLT provides essential guidelines for designing the learning experience, highlighting the importance of managing intrinsic cognitive load and reducing extraneous load to optimize information processing and effective learning.

### 2.2. Gamification Approach

In this study, the intervention is framed within gamification, broadly defined as the application of game elements to non-game contexts to foster learning and engagement. Within this framework, serious games (SGs) represent a specific subset, as they are full game-based environments explicitly designed for educational purposes. Accordingly, we use the term SG when referring to the developed tool, while maintaining the broader notion of gamification as the theoretical underpinning.

The SG developed for this study was created using the Quizziz platform (now WayGround) and consisted of a sequence of predetermined texts and images embedded within multiple clinical scenarios. Within these scenarios, several interactive multiple-choice questions were included to guide participants’ attention and reasoning toward key clinical information progressively revealed throughout the narrative. Correct answers were awarded points, and each response, whether correct or incorrect, was followed by written immediate feedback explaining the rationale behind it. In all cases, however, the game’s narrative path remained pre-defined. The structure of the SG followed a simple and intuitive format widely employed in the literature and comparable to existing examples of virtual simulation in Italy, such as SIMMED Connect.

**Figure 1:**
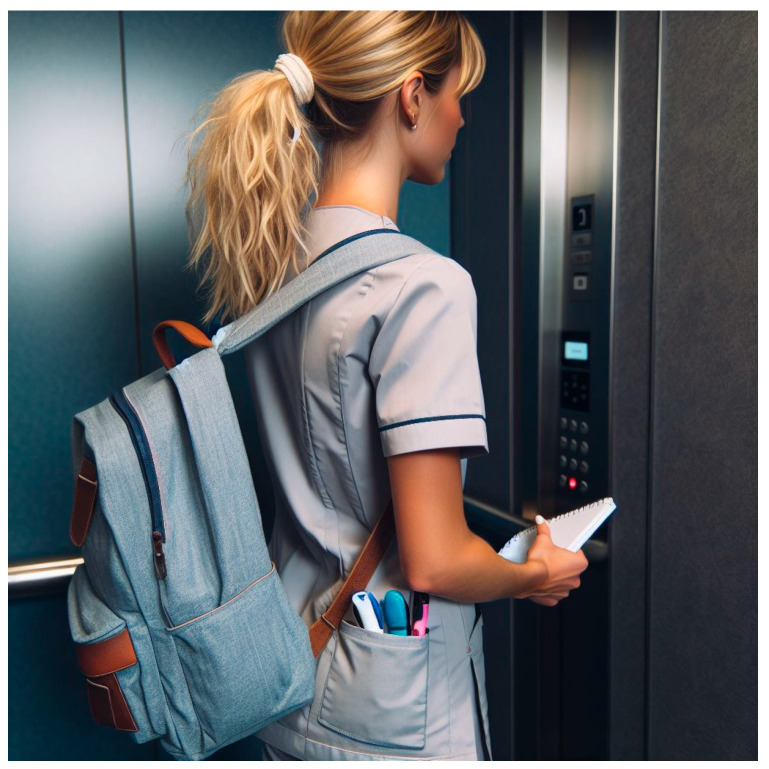
Beatrice exiting the elevator at the entrance to the psychiatry ward.

**Figure 2:**
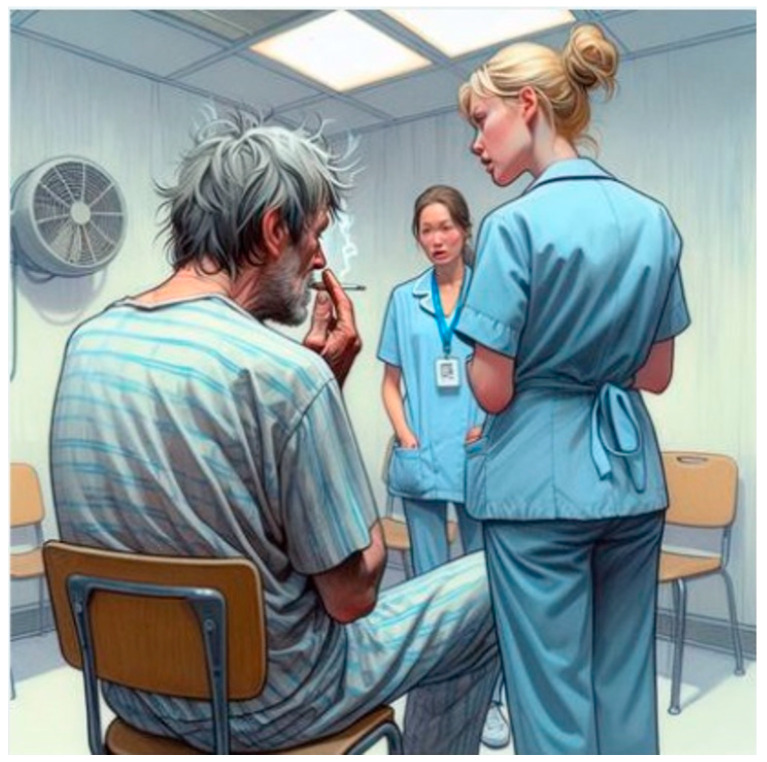
Beatrice and a Nurse Assistant with the patient Carmelo in the Smoking Room.

**Figure 3:**
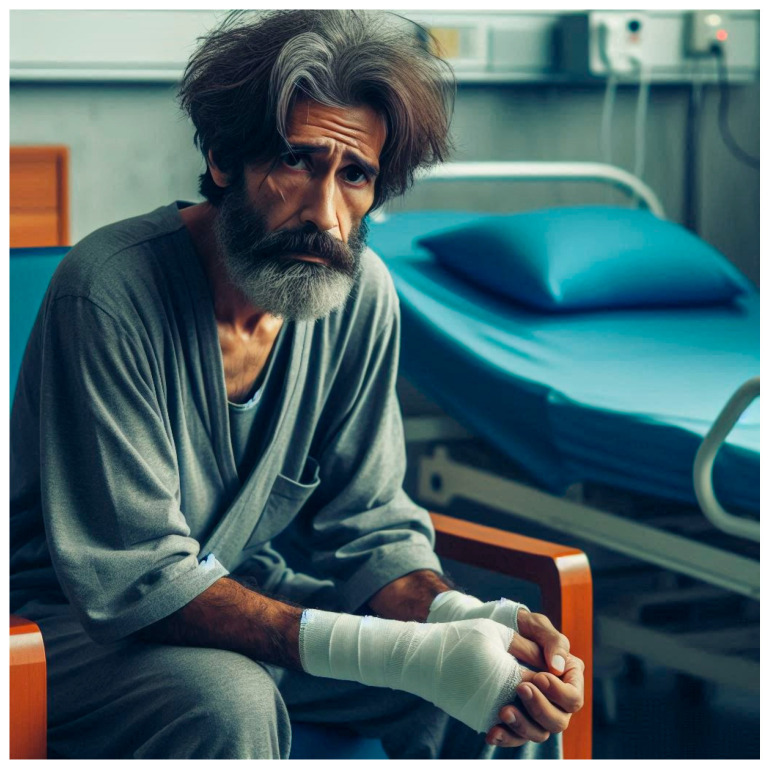
Patient Massimo as seen by Beatrice upon entering his room.

Through the combination of textual content and interactive questions, the game delivered theoretical knowledge and engaged students in practicing two separate nursing handovers (each for a different patient) using the SBAR method. All visual elements were generated using Microsoft Copilot AI (version GPT4) and served solely to support the atmosphere of the scenarios. These images were not intended to convey clinical data or influence decision-making. At the beginning of the game, a disclaimer informed participants not to consider the images as clinically relevant components of the simulation. Figure 1, Figure 2 and Figure 3 shows different representative screens of the serious game.

### 2.3. Sample

A voluntary convenience sample of 48 undergraduate nursing students was recruited from a university in Northern Italy, including 29 second-year and 19 third-year students. At the time of data collection, all participants had completed clinical placements in medical and surgical units. However, only four students had previous training experience in mental health settings (e.g., Psychiatric Diagnosis and Care Services, SPDC). Theoretical coursework on clinical nursing in neuropsychiatric disabilities had already been completed. All students were previously introduced to the structure and components of the SBAR communication framework.

The sample size was calculated a priori using G*Power (version 3.9.1.7) based on a two-tailed *t*-test for two independent samples, assuming a plausible moderately large effect size (*d* = 0.6) due to the immediate post-intervention measurement, a significance level of α = 0.05, and a statistical power of 0.80 (1 − β). The analysis indicated that a minimum of 45 participants per group would be required to detect a statistically significant effect.

### 2.4. Scenario Context

The scenarios included in the SG were set within a Psychiatric Diagnosis and Care Unit and were specifically designed to emphasize the importance of structured information transfer and communication methodology during nursing handovers, rather than focusing on detailed clinical knowledge of the cases themselves. The SG followed the experience of Beatrice, a second-year nursing student undertaking her clinical placement in a psychiatric ward, as she provided care to two main patients:

Massimo, a patient diagnosed with major depressive disorder, admitted under compulsory treatment (TSO) following a suicide attempt;

Carmelo, a patient with a prior diagnosis of schizophrenia, admitted due to auditory hallucinations.

Other characters that featured in the scenarios included Beatrice’s clinical preceptor, Mairo (a registered nurse), and two nurse assistants.

### 2.5. Content Validity

Prior to its implementation and digital adaptation, the content of the SG underwent a global content validity assessment [34] conducted by a panel of six expert nurses, each with more than 10 years of experience in educational settings. One of the experts also had over 10 years of clinical experience in mental health care. Based on the feedback received, the scenario content was revised accordingly. Following this revision, a second evaluation confirmed that the SG content was appropriate and valid for the intended educational objectives.

### 2.6. Pre–Post Test

The pre–post test was specifically developed for this educational intervention and consisted of three sections. The first section collected sociodemographic information, including age, gender, academic year repetition, prior qualification as a health care assistant, and previous attendance at lectures or workshops specifically focused on nursing handovers. The second and third sections comprised 26 statements derived from two realistic nursing handover scenarios: one from a surgical context (14 statements) and one from a medical context (12 statements). Students were asked to reorganize the information presented in the 26 statements as if they were delivering a clinical handover using the SBAR communication framework. Each test response was scored dichotomously, with 1 point assigned for a correct answer and 0 points for an incorrect answer. The maximum total score was therefore 26. The 26 items were distributed across the SBAR domains as follows: Situation = 6 items, Background = 4 items, Assessment = 11 items, and Recommendation = 5 items. The pre–post test underwent content validity assessment by the same expert panel that had previously evaluated the content of the SG, confirming its immediate validity; the panel also reviewed the test items and agreed on the correct answers for each. Following administration, internal consistency analyses were performed by calculating Cronbach’s alpha and McDonald’s omega, both for the overall scale and through item-deleted analysis. Both coefficients indicated the excellent reliability of the instrument in the pre- (α = 0.848; ω = 0.855) and the post-test (α = 0.812; ω = 0.827) phases. Item deletion did not produce any substantial variation in reliability estimates.

The decision to use clinical scenarios from medical and surgical settings, despite the educational intervention being set in a mental health context, was driven by the objective of assessing not the memorization or mechanical reproduction of information practiced during the game, but rather the actual learning, transferability, and ability to apply the SBAR framework. Since SBAR is a cross-contextual communication tool, testing students with clinical cases different from those used in the SG allowed for evaluating deeper, more generalizable learning outcomes, in line with Kolb’s Experiential Learning Theory [29] by encouraging the abstract conceptualization and active experimentation phases beyond the original learning context, with Cognitive Load Theory [32] principles by minimizing context-specific cues and promoting schema transfer, and with Self-Determination Theory [30,31] by fostering competence through the demonstration of adaptable communication skills across varied clinical contexts. Moreover, all participants had already completed clinical placements in medical and surgical units, whereas not all had been exposed to psychiatric settings (e.g., Psychiatric Diagnosis and Care Services, SPDC). The use of scenarios from more familiar contexts, therefore, minimized potential bias due to unequal practical exposure and ensured a more equitable evaluation of the intervention’s effectiveness. The complete text of the test is available in Appendix A.

### 2.7. Procedure

The pre-test questionnaire was administered and collected in person by the researchers prior to the gamified educational experience. Following the questionnaire, each student accessed the SG intervention individually via a link, using either a tablet or computer to facilitate readability (while the content was accessible on smartphones, this option was discouraged due to the limited screen size). Upon completing the gamification activity, which lasted approximately 45 min, students were administered the post-test, which was identical in content to the pre-test. At the end of the experience, a group debriefing session was conducted with all participants, lasting approximately ten minutes.

### 2.8. Student Experience

The experience with the SG was evaluated using the Player Experience Inventory (PXI), a scale previously adopted in the literature to assess nursing students’ experiences with SGs in educational settings [35]. The PXI consists of 33 items scored on a −3 to +3 Likert scale, where higher scores indicate stronger agreement with positive statements. Thus, values above 0 reflect a neutral experience, with increasing scores representing progressively better evaluations. This tool, originally developed by Professor Vanden Abeele [36], was translated into Italian using the back-translation method [37]. The translation process involved two independent translations performed by nursing researchers with a certified C2 level of English proficiency. In cases of discrepancy between the two versions, the research team selected the translation that best fit the Italian cultural context. The Italian translation was subsequently back-translated and returned to the original author for conceptual and semantic equivalence verification. Reliability analyses, performed using Cronbach’s alpha and McDonald’s omega, demonstrated excellent internal consistency (α = 0.90, ω = 0.908). The removal of individual items did not produce any notable changes in reliability coefficients.

### 2.9. Statistical Analysis

Statistical analysis was performed with statistical software R V.4.5.1 and JAMOVI V.2.6.23.

### 2.10. Ethical Considerations and Approvals

This study received formal approval from the Academic Director and the Internal Review Board of the Bachelor School of Nursing where the intervention was conducted (Authorization No. PF/MP025, dated 25 November 2024). All participants provided written informed consent prior to participation. It is important to note that, under Italian law, the educational methodology employed in this study falls within the scope of standard academic discretion. Aside from the collection of written informed consent, no personally identifiable information from individual students was recorded.

## 3. Results

After obtaining authorizations, ninety-six tests were collected and analyzed (48 pre-tests and 48 post-tests), completed by a sample of 48 nursing students, comprising 29 s-year and 19 third-year students. The sample was predominantly female (77.1%) and included a high proportion of students who were repeating the academic year (62.5%). The mean age was 23.2 years old (SD ± 5.1), with a range from 19 to 49 years old. Table 1 presents the sociodemographic characteristics of the sample.

**Figure 4:**
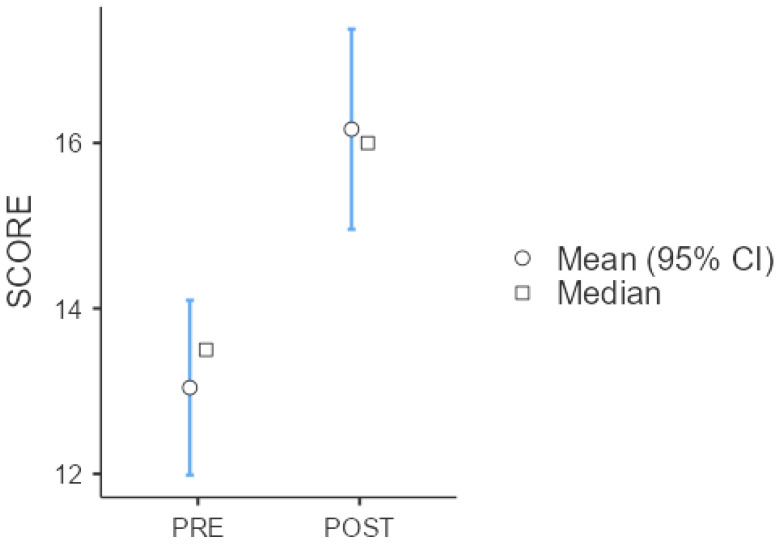
Score Descriptives Statistic.

Since 35.4% of students (n = 17) reported no prior formal training in handover methodologies, we compared their performance with trained peers. Assumptions of normality (Shapiro–Wilk test = 0.989, *p* = 0.587) and homogeneity of variance (Levene’s test: F = 1.50, *p* = 0.223) were met. An independent samples *t*-test revealed no statistically significant difference between the two groups (Student’s *t*-test = 0.369, *p* = 0.713). The normality of the overall score distribution (Shapiro–Wilk test = 0.986, *p* = 0.389) and the homogeneity (Levene’s test = 0.816, *p* = 0.369), allowed for an independent samples *t*-test to compare pre- and post-intervention scores. The analysis indicated a statistically significant improvement following the educational intervention (Student’s *t*-test = −3.81, *p* < 0.001), with a moderate effect size (Cohen’s d = −0.778). The results are summarized in Table 2 and Figure 4.

The learners’ gaming experience was assessed using the Italian version of the Player Experience Inventory (PXI). Due to partial completion of some questionnaires, only 41 fully completed forms out of the 48 distributed (85.42%) were included in the analysis. Although Bartlett’s test of sphericity yielded statistically significant results (χ^2^ = 1400, *p* < 0.001), the small sample size and low Kaiser–Meyer–Olkin (KMO) value (0.496) indicated insufficient sampling adequacy, thus precluding the use of factor analysis via either EFA or PCA. When PXI scores were analyzed based on negative (−1, −2, −3), positive (1, 2, 3), and neutral (0) values, the overall experience was reported as predominantly positive by the majority of students across the domains of Meaning, Curiosity, Progress Feedback, Audiovisual Appeal, Challenge, Ease of Control, Clarity of Goal, and Enjoyment. A summary of the results is provided in Table 3.

The Immersion domain showed mostly positive responses for items 14 and 15; however, item 13 (“*I wanted to explore how the game evolved*”) revealed a near-even distribution of negative (43.8%) and positive (46.3%) ratings, with 9.8% of students responding neutrally. The domains of Mastery and Autonomy (items 7–13) showed a general tendency toward negative responses, except for item 10 (“*I felt free to play the game in my own way*”), which received positive ratings from 58.5% of participants. A comparison of PXI item scores between second- and third-year students using the Mann–Whitney U test revealed no statistically significant differences, with the exception of item 9 (Mastery—“I was fully focused on the game”) and two items from the Immersion domain (item 14—“I wanted to find out how the game progressed”; item 15—“I felt eager to discover how the game continued”). Nevertheless, given that the factor structure of the PXI was not supported in our sample (KMO < 0.6), these item-level differences should be interpreted cautiously, as they cannot be ascribed to psychometrically validated subscales. Full results are reported in Table S1. 

A comparison was conducted between selected PXI items reported in previous studies involving SGs for nursing education [35] and those obtained in this study. For this purpose, response values were re-coded on a 7-point Likert scale to enable direct comparison. Independent samples *t*-tests were then performed for each item using the mean scores, standard deviations, and sample sizes to allow for a crude comparative analysis (in Table 4).

Although it was possible to compare only 10 out of the 33 full PXI items, the results revealed statistically significant differences in the domains of Mastery, Immersion, Autonomy, and Progress Feedback. An analysis of the response trends using correlation tests yielded significant values for both Spearman’s rho (ρ = 0.867, *p* = 0.003) and Kendall’s tau-b (τb = 0.733, *p* = 0.002), indicating a consistent pattern in score variation across the two samples.

## 4. Discussion

This study aimed to assess the effectiveness of an educational intervention designed to support nursing students in learning the SBAR methodology, a structured communication tool, through an SG scenario set in a mental health care context, consistent with approaches previously reported in the literature [24].

Although the majority of participants (62.5%) reported having previously attended lectures on structured handover methods, and the nursing curriculum formally stated that these lessons specifically addressed SBAR, no statistically significant differences were observed between the scores of students who had received such instruction and those who had not. Conversely, the proposed SG intervention yielded statistically significant improvements in pre- and post-test scores, underscoring the effectiveness of the educational strategy, with a large effect size (Cohen’s d = −0.78) that indicates not only statistical, but also educationally and clinically meaningful improvement. In practical terms, this effect reflects a substantial enhancement of students’ ability to apply the SBAR framework after the intervention, which is likely to translate into more structured, accurate, and safer handovers in clinical training contexts. These findings not only support the value of the SG-based intervention but also suggest that it may be equally or even more effective than traditional didactic instruction.

From a theoretical perspective, this result aligns with Kolb’s Experiential Learning Theory, as the SG actively engaged learners in a cycle of concrete experience, reflective observation, abstract conceptualization, and active experimentation processes, less prominent in passive instructional formats. It is also consistent with Self-Determination Theory, which emphasizes that satisfying learners’ needs for autonomy, competence, and relatedness fosters intrinsic motivation and engagement. By allowing students to actively apply the SBAR framework in an interactive environment, the SG may have promoted a stronger sense of competence and ownership over the learning process. Finally, Cognitive Load Theory could suggest that the SG’s design, with guided choices and scenario-based learning, helped to manage intrinsic cognitive load while reducing extraneous load, thus facilitating schema acquisition and transfer. In combination, these mechanisms provide a theoretical basis for understanding why the SG format can match or exceed the educational impact of traditional instruction.

However, the absence of follow-up assessments (e.g., evaluations conducted one month after classroom teaching and one month after the SG-based intervention) limits the ability to draw definitive conclusions about long-term retention. Nevertheless, this possibility should not be dismissed. Previous studies have shown that gamified interventions in nursing education can, in some cases, outperform conventional teaching methodologies in terms of educational outcomes [19,20,21,22,23,24,38].

As reported in other studies [35], students’ experience, as measured by the PXI, was overall positive across the domains of *Meaning*, *Curiosity*, *Ease of Control*, *Challenge*, *Progress Feedback*, *Audiovisual Appeal*, *Clarity of Goals*, and *Enjoyment*, with the exception of items 7, 8, 9, 11, 12, and 13, which belong to the domains of *Autonomy*, *Mastery*, and *Immersion*.

Although these values were negatively oriented, several practical and design-related considerations should be taken into account. Regarding Autonomy, the structure of the game did not allow for full decisional or gameplay independence, as the available choices were guided and predetermined to ensure alignment with the intended learning outcomes. While this approach may have limited perceived autonomy, it was consistent with the instructional goal of directing students toward correct SBAR usage. It is also worth noting that, given the widespread diffusion of open-world and highly interactive digital games, younger students are increasingly accustomed to experiencing unrestricted freedom in virtual environments; therefore, they may have perceived the structured and goal-oriented nature of our game as more limiting in terms of autonomy. For Mastery, considering that this was the students’ first exposure to such an SG, it is reasonable to assume that they had not yet developed sufficient familiarity with the interface or gameplay mechanics to feel fully competent or in control. This initial learning curve may have influenced their self-assessment in this domain and suggests that repeated exposure or progressive levels of complexity might enhance future Mastery scores. Moreover, it is plausible that students who realized they had previously misclassified or omitted information during the handover task may have rated themselves lower on Mastery, as the awareness of mistakes can temporarily reduce perceived competence despite contributing to learning. From the perspective of Self-Determination Theory, this finding aligns with the notion that perceived competence is highly sensitive to immediate performance feedback: while errors may undermine short-term perceptions of mastery, they can also represent valuable opportunities for skill development and growth when supported by iterative practice. This interpretation is consistent with our initial hypothesis regarding the mechanism of gamification, whereby structured feedback and the opportunity to recognize and correct errors may transiently lower perceived mastery but ultimately foster deeper learning and skill consolidation.

In the Immersion domain, a statistically significant difference emerged between second- and third-year students, which could reflect a developmental trajectory in which more advanced students focus more on the learning process than on the surrounding context. Within this domain, item 13 was the only one to show a non-positive trend, with responses almost evenly split between positive (approximately 46%) and negative (approximately 43%) ratings. This result may be partly attributed to the SG’s design, which relied on written narrative and static images. While this format can be engaging, it is relatively simple. It requires cognitive effort for reading and comprehension—factors that may not align with the preferences of newer generations, who often favor fast-paced, multimedia-rich formats that demand less processing effort [39]. This interpretation is further supported by the significantly lower Immersion scores observed in our study compared with an SG developed using more complex, unfolding video scenarios [35].

Although both interventions shared a common structure, namely unfolding scenarios with multiple choices for progression, the use of video-based unfolding scenarios may offer greater accessibility and help students to feel more confident in their ability to engage with the game, as supported by other findings in the literature [40]. Such scenario also partially explains the significant difference observed in the Mastery domain. It is important to note, however, that the scores considered are raw and that the student samples in the two studies belonged to very different cultural and geographical settings. Finally, the correlation observed between the scores of the video-based SG and the SG used in this study suggests the presence of a common pattern in students’ experiences, regardless of the SG format adopted. The similarity in response trends may suggest that the experiential construct measured is influenced not only by the mode of delivery (e.g., video format vs. text and image format) but also by other educational design elements such as scenario structure, limited autonomy due to predetermined choices, and technical limitations of the online platforms used (e.g., restricted feedback mechanisms), as reported in previous studies [41,42]. Nonetheless, it is noteworthy that none of the students assigned a negative value to the SG in terms of its overall relevance (item 2), consistently acknowledging its educational value.

Finally, only a small percentage of students (4–7%) reported not enjoying the experience or not liking the gameplay (external domain: Enjoyment). As reported in previous experiences described in the literature [43,44], the previously identified lack of autonomy embedded in the design (e.g., limited decision-making power) may have influenced the results for those items. Additionally, these figures may reflect students who encountered difficulties engaging with the game due to personal characteristics. For instance, it is plausible that some may have had specific learning needs, such as dyslexia, which could have made this instructional format more challenging without an adaptive version of the game [45,46]. Finally, considering the high proportion of repeating students within the sample, some underperforming students, characterized by different learning trajectories, may have needed to exert greater effort than their peers to complete the game.

### 4.1. Limitations

Although the effect size indicated a moderate impact of the SG-based educational intervention and the literature increasingly supports the use of gamification, the conclusions drawn from this study remain limited. Among the main limitations are the small sample size and the use of a non-probabilistic convenience sampling method, the monocentric design, and the inability to assess the long-term retention of the learning outcomes. Moreover, the absence of a control group prevents any objective comparison with other instructional methods that may have been used for similar educational purposes; causal inferences are inherently limited by the one-group design. Furthermore, potential testing effects due to repeated exposure to the same instrument and the possibility of social desirability bias (whereby participants may have provided responses that they believed were expected) should be acknowledged. Given the short time interval between pre- and post-test administration, maturation effects are unlikely to have influenced the results. Additionally, the debriefing was conducted after the post-test, which may have reduced SG’s potential educational impact. Another major methodological limitation lies in the absence of a matching system between pre- and post-intervention tests at the individual level. Although the quasi-experimental pre–post design allowed for detecting significant aggregate differences, the lack of paired data limited the possibility of assessing individual improvements and conducting more robust statistical analyses (e.g., regression models, paired data tests, subgroup analyses). For future studies, using unique anonymous codes is recommended to enable longitudinal analyses without compromising participant confidentiality. While the gamification format employed in this study appears promising, further investigation is warranted, and a randomized controlled trial comparing lecture- and video-based SG training is necessary.

### 4.2. Implications for Nursing Education and Research

From a practical perspective, these findings indicate that SGs can be readily integrated into the teaching of SBAR within mental health nursing curricula. They may serve both as preparatory activities before clinical placements and as safe environments in which students can consolidate communication skills without exposing patients to risk. The significant improvement in post-test scores compared to pre-test results, combined with the positive learner experience as measured by the PXI, indicates that this approach may represent a valid alternative or a valuable complement to traditional face-to-face instruction. In light of these results, it is essential to further investigate the long-term effectiveness of such tools through time-based follow-ups aimed at assessing the stability of acquired SBAR competencies. Additionally, multisite randomized controlled trials comparing lecture-based SGs with other instructional methods (e.g., video-based SGs or Virtual Realities) would help to identify the most effective strategies and enhance the generalizability of the findings. Future research should also explore alternative game formats, such as interactive videos or virtual reality, which may enhance immersion and improve perceptions of Autonomy and Mastery, domains that appeared more critical in this study. Moreover, the applicability of the SG should be evaluated in different clinical contexts and with diverse student populations. The adoption of more sophisticated methodological tools, such as individual-level pre–post matching and subgroup analyses, may contribute to a deeper understanding of learning dynamics and support the adaptation of educational interventions to specific learner needs. Finally, it is crucial to assess the accessibility and usability of the game for students with special educational needs, ensuring inclusiveness and equal learning opportunities. Well-designed SGs can serve as effective learning tools for students with specific learning disorders, providing interactive, multisensory, and adaptive experiences that may facilitate skill acquisition and help learners to achieve their educational goals more efficiently. Recent evidence suggests that SGs could improve reading and phonological skills in children with dyslexia, while increasing motivation and enjoyment in the learning process [47]. Similarly, a feasibility study of a rhythm-based SG specifically developed for children with dyslexia demonstrated promising usability and potential for aiding literacy development [48]. Although no official national or international estimates are currently available, emerging evidence suggests that the proportion of nursing students with specific learning disorders may reach up to 12.5% [49], with a tendency to increase over time. In this light, the purposeful integration of SGs into nursing curricula could represent a forward-looking and impactful strategy, opening a new frontier in the effective education and inclusion of this student population’s needs. Finally, considering that a large proportion of our sample consisted of repeating students, gamification could also be strategically employed to keep previously acquired skills and knowledge ‘alive,’ even at a distance, thus supporting continuity of learning and reducing the decay of communication competencies over time.

In the long term, the structured integration of gamification into undergraduate nursing curricula should be considered a strategic goal, supported by scientific evidence and ongoing validation.

## 5. Conclusions

This study demonstrated the effectiveness of an SG, set in a mental health care context, as an educational tool to improve nursing students’ communication skills in structured handover using the SBAR model. The pre–post comparison revealed a statistically significant improvement in performance, supporting the value of game-based learning even within complex educational domains. Although some challenges were noted in the domains of Autonomy, Immersion, and Mastery, students’ subjective experience was largely positive, indicating high levels of acceptability and satisfaction with the method. Nonetheless, key limitations, including the single-center design, small sample size, absence of a control group, and lack of individual-level pre–post matching, limit the generalizability of the findings. Further research employing more rigorous methodological designs is warranted to strengthen the evidence base regarding the effectiveness of gamification in nursing education, particularly in clinical areas characterized by high communication demands, such as mental health.

Ultimately, this study contributes to filling a gap in the existing literature and opens new avenues for integrating innovative teaching strategies into the training of future health care professionals, reinforcing the essential link between technical competence and clinical sensitivity, both of which are critical for ensuring quality and safety in nursing care.

## Figures and Tables

**Table 1:** Socio-demographic characteristics of the sample.

Gender	
Female	37 (77.1%)
Male	10 (20.8%)
Prefer not to disclose	1 (2.1%)
Age	
Mean (SD)	23.2 (5.1)
Range	19.0–49.0
Missing	1
Repeater Student	
Yes	30 (62.5%)
No	18 (37.5%)
Nurse Assistant Qualification	
Yes	2 (4.17%)
No	46 (95.83%)
Attended Handover Lessons	
Yes	31 (64.6%)
No	17 (35.4%)

**Table 2:** Test Score Descriptives and *t*-test.

Group Descriptives						
	**Group**	**N**	**Mean**	**Median**	**SD**	**SE**
SCORE	PRE	48	13	13.5	3.74	0.539
	POST	48	16.2	16	4.28	0.618
**Independent samples *t*-test**		**Statistic**	**Df**	***p***	**Effect Size**	
	Student’s t	**−3.81**	**94**	**<0.001**	Cohen’s d	**−0.78**
	Mann–Whitney U	**676**		**<0.001**	Rank biserial correlation	**0.414**

Statistically significant values are in bold.

**Table 3:** Summarized PXI Score.

DOMAIN	Item	Negative (%)	Neutral (%)	Positive (%)	Mean	SD
Meaning	1	2.45	19.5	78.05	1.71	1.23
	2	0.00	9.8	90.24	2.12	0.98
	3	4.91	14.6	80.49	1.63	1.24
Curiosity	4	12.15	9.8	78.05	1.29	1.42
	5	9.73	4.9	85.37	1.37	1.36
	6	17.09	19.5	63.41	0.88	1.54
Mastery	7	34.13	36.6	29.27	−0.10	1.59
	8	41.48	19.5	39.02	−0.07	1.52
	9	53.69	26.8	19.51	−0.66	1.51
Autonomy	10	24.36	17.1	58.54	0.56	1.72
	11	46.37	26.8	26.83	−0.20	1.69
	12	36.57	36.6	26.83	−0.17	1.69
Immersion	13	43.86	9.8	46.34	−0.12	2.05
	14	19.51	12.2	68.29	0.90	1.77
	15	19.52	19.5	60.98	0.80	1.57
Progress Feedback	16	12.23	14.6	73.17	1.37	1.48
	17	12.15	9.8	78.05	1.37	1.3
	18	21.93	4.9	73.17	1.37	1.73
Audiovisual Appeal	19	2.42	4.9	92.68	1.85	1.06
	20	4.92	2.4	92.68	2.00	1.07
	21	2.40	9.8	87.80	1.90	1.16
Challenge	22	2.40	9.8	87.80	1.61	1.05
	23	4.85	17.1	78.05	1.22	1.11
	24	7.35	26.8	65.85	1.12	1.17
Ease of Control	25	9.71	9.8	80.49	1.68	1.33
	26	4.92	2.4	92.68	1.90	1.09
	27	2.43	12.2	85.37	1.88	1.08
Clarity of Goals	28	2.42	4.9	92.68	2.29	0.87
	29	2.46	7.3	90.24	2.07	1.01
	30	2.46	7.3	90.24	2.07	1.01
Enjoyment	31	4.90	7.3	87.80	1.80	1.33
	32	7.33	7.3	85.37	1.51	1.31
	33	7.27	9.8	82.93	1.56	1.38

Colours indicate the division between psychosocial consequences (light blue), the functional consequences (light green) and enjoyment (orange).

**Table 4:** PXI Results Comparison.

Survey Items (Constructs)	Video-Based SG [35] Score (SD)	This SG Score (SD)	Independent *t*-Test	*p*-Value
**Psychosocial consequences**				
Playing the game was meaningful to me (Meaning)	5.99 (1.32)	5.71 (1.23)	1.3175	0.1882
I felt I was good at playing this game (Mastery)	5.23 (1.45)	3.9 (1.59)	5.6346	**< 0.0001**
I was immersed in the game (Immersion)	5.78 (1.39)	4.9 (1.77)	3.8372	**0.0001**
I felt free to play the game in my own way (Autonomy)	5.44 (1.65)	4.76 (1.71)	2.5423	**0.0113**
I wanted to explore how the game evolved (Curiosity)	5.70 (1.44)	5.29 (1.41)	1.7631	0.0784
**Functional consequences**				
I thought the game was easy to control (Ease of Control)	5.88 (1.42)	5.88 (1.07)	0	1
The game was not too easy and not too hard to play (Challenge)	5.46 (1.61)	5.61 (1.04)	0.5875	0.5571
The game gave clear feedback on my progress towards the goals (Progress feedback)	5.98 (1.31)	5.37 (1.72)	2.8132	**0.0051**
I enjoyed the way the game was styled (Audiovisual appeal)	6.02 (1.38)	5.9 (1.15)	0.5432	0.5872
The goals of the game were clear to me (Goals and rules)	6.11 (1.26)	6.07 (1.1)	0.1979	0.8432

Statistically significant values are in bold.

## Data Availability

The original data presented in the study are openly available in Mendeley Data at https://doi.org/10.17632/vtr6gbzxxd.1 (accessed on 12 April 2025).

## Supplementary Materials

The following supporting information can be downloaded at: https://www.mdpi.com/article/10.3390/nursrep15090322/s1, Table S1: Complete PXI results.

## Public Involvement Statement

No public involvement in any aspect of this research.

## Guidelines and Standards Statement

This manuscript was drafted against the TREND (Transparent Reporting of Evaluations with Nonrandomized Designs) statement for nonrandomized research.

## Abbreviations

The following abbreviations are used in this manuscript:

SG	Serious Game
SBAR	Situation, Background, Assessment, Recommendation
SDT	Self-Determination Theory
CLT	Cognitive Load Theory

## Appendix A. Test Sentences to Be Reorganized According to the SBAR Method (English Translation and Italian Original Version)

### Appendix A.1. English Version

Internal Medicine Case
  ☐ During the ward round, the patient reports burning at the catheter insertion site.
  ☐ Five days ago, Mr. Giovanni, 79 years old, presented to the Emergency Department (ED) accompanied by his son.
  ☐ The patient has had arterial hypertension for 15 years and COPD for the past 4 years. Giovanni has smoked 20 cigarettes/day for 40 years.
  ☐ Blood and catheter urine cultures were collected at 2:30 p.m. following a physician’s order.
  ☐ Giovanni lives alone.
  ☐ He arrived at the ED for dyspnea with SpO_2_ at 91% on room air and peripheral edema. Oxygen therapy was administered according to medical prescription.
  ☐ Today, broad-spectrum antibiotic therapy was initiated (Amoxicillin and Clavulanic Acid).
  ☐ At 3:00 p.m., the following vital signs were recorded: BP 130/75 mmHg, HR 98 bpm, SpO_2_ 95% on oxygen therapy, no dyspnea at rest, RR 22/min, tympanic temperature consistently 38.5 °C.
  ☐ Temperature should be monitored.
  ☐ Admitted to the Internal Medicine Unit for acute heart failure.
  ☐ Furosemide 20 mg IV was administered at 4:00 p.m.
  ☐ Check the results of the culture tests.

surgical case
  ☐ One year ago, the patient was diagnosed with arterial hypertension, and seven years ago with type 2 diabetes mellitus. At home, she takes Amlodipine 5 mg and Metformin 500 mg.
  ☐ At triage, she reported severe abdominal pain in the right iliac fossa, NRS 6/10.
  ☐ Assess the need for enema administration.
  ☐ Pain is currently under control, NRS 2/10.
  ☐ Since admission to the ward, the patient has not had a bowel movement, including during the current shift.
  ☐ The patient has a urinary catheter, which self-detached during the shift.
  ☐ The surgical wound is clean and dry, with no signs of infection.
  ☐ Mrs. Clara reports occasional nausea.
  ☐ No known allergies.
  ☐ Admitted to the Surgical Unit for laparoscopic appendectomy performed three days ago.
  ☐ Reassess the surgical wound.
  ☐ At 9:00 a.m., the following vital signs were recorded: BP 110/70 mmHg, HR 78 bpm, SpO_2_ 97% on room air.
  ☐ Mrs. Clara, 53 years old, presented to the ED accompanied by her husband.
  ☐ Verify that the patient resumes spontaneous urination.

### Appendix A.2. Italian Version

Caso internistico:
  ☐ Al giro visita l’assistito riferisce bruciore all’inserzione del catetere
  ☐ Cinque giorni fa il signor Giovanni di 79 anni si presenta in PS accompagnato dal figlio
  ☐ Da 15 anni il paziente soffre di ipertensione arteriosa e da 4 anni soffre di BPCO. Giovanni fuma 20 sigarette/die da 40 anni
  ☐ Sono state eseguite emocolture ed urinocolture da catetere su prescrizione medica alle ore 14:30
  ☐ Giovanni vive da solo.
  ☐ Giunge in PS per dispnea con SpO2 91% in AA e edemi declivi. Si somministra O2tp come da prescrizione medica.
  ☐ Oggi ha iniziato la terapia con antibiotico ad ampio spettro (Amoxicillina e Acido Clavulanico)
  ☐ Alle ore 15:00 oggi presentava i seguenti PV: PA 130/75 mmHg, FC 98 bpm, SpO2 95% in O2tp, assenza di dispnea a riposo, FR 22 a/min, TC sempre 38.5 °C (timpanica).
  ☐ Va monitorare la TC
  ☐ Ricoverato nell’UO di Medicina Interna per scompenso cardiaco acuto
  ☐ È stato somministrato Furosemide 20 mg EV h 16.00
  ☐ Verificare l’esito degli esami colturali

Caso Chirurgico
  ☐ Un anno fa le è stata diagnosticata l’ipertensione arteriosa e 7 anni fa il DM2. Al domicilio l’assistita assume Amlodipina 5 mg e Metformina 500 mg
  ☐ Al triage riferisce forti algie addominali in fossa iliaca destra, NRS 6/10
  ☐ Valutare esecuzione di clistere
  ☐ Dolore controllato, attualmente NRS 2/10
  ☐ Dall’ingresso in reparto l’assistita non si è ancora scaricata, nemmeno durante il turno
  ☐ L’assistita ha un CV che nel corso del turno si auto-rimuove
  ☐ La ferita è pulita e asciutta, non è infetta
  ☐ La signora Clara riferisce nausea occasionale
  ☐ Non allergie note
  ☐ Ricoverata nell’UO di Chirurgia per appendicectomia laparoscopica 3 giorni fa’
  ☐ Rivalutare la ferita chirurgica
  ☐ Alle ore 9:00 si rilevano i seguenti PV: 110/70 mmHg, 78 bpm, 97% AA
  ☐ La signora Clara di 53 anni si presenta in PS accompagnata dal marito
  ☐ Verificare che l’assistita riprenda ad urinare spontaneamente
