# Supplementary material for: Effectiveness of a Gamification-Based Intervention for Learning a Structured Handover System Among Undergraduate Nursing Students: A Quasi-Experimental Study
Source: Nurs Rep. 2025 Sep 4;15(9):322. doi: 10.3390/nursrep15090322 (PMC12472369; doi:10.3390/nursrep15090322)
Supplement: Supplementary file 1 [file nursrep-15-00322-s001.zip › nursrep-3800607-supplementary.pdf]

Supplementary Table S1. Complete PXI Results.

Items	n. (%)						
	-3	***-2***	***-1***	***0***	***1***	***2***	***3***
Playing the game was meaningful to me.	-	1 (2.4%)	-	8 (19.5%)	4 (9.8%)	16 (39%)	12 (29.3%)
The game felt relevant to me.	-	-	-	4 (9.8%)	5 (12.2%)	14 (34.1%)	18 (43.9%)
Playing this game was valuable to me.	-	1 (2.4%)	1 (2.4%)	6 (14.6%)	7 (17.1%)	15 (36.6%)	11 (26.8%)
I felt I was good at playing this game.	1 (2.4%)	1 (2.4%)	3 (7.3%)	4 (9.8%)	10 (24.4%)	15 (36.6%)	7 (17.1%)
I felt capable while playing the game.	1 (2.4%)	1 (2.4%)	2 (4.9%)	2 (4.9%)	15 (36.6%)	12 (29.3%)	8 (19.5%)
I felt a sense of mastery playing this game.	2 (4.9%)	-	5 (12.2%)	8 (19.5%)	12 (29.3%)	7 (17.1%)	7 (17.1%)
I was no longer aware of my surroundings while I was playing.	4 (9.8%)	4 (9.8%)	6 (14.6%)	15 (36.6%)	3 (7.3%)	8 (19.5%)	1 (2.4%)
I was immersed in the game.	3 (7.3%)	4 (9.8%)	10 (24.4%)	8 (19.5%)	8 (19.5%)	8 (19.5%)	-
I was fully focused on the game.	5 (12.2%)	8 (19.5%)	9 (22%)	11 (26.8%)	3 (7.3%)	5 (12.2%)	-
I felt free to play the game in my own way.	2 (4.9%)	5 (12.2%)	3 (7.3%)	7 (17.1%)	12 (29.3%)	6 (14.6%)	6 (14.6%)
I felt like I had choices regarding how I wanted to play this game.	3 (7.3%)	8 (19.5%)	6 (14.6%)	11 (26.8%)	7 (17.1%)	2 (4.9%)	4 (9.8%)
I felt a sense of freedom about how I wanted to play this game.	3 (7.3%)	8 (19.5%)	4 (9.8%)	15 (36.6%)	4 (9.8%)	3 (7.3%)	4 (9.8%)
I wanted to explore how the game evolved.	7 (17.1%)	7 (17.1%)	4 (9.8%)	4 (9.8%)	9 (22%)	5 (12.2%)	5 (12.2%)
I wanted to find out how the game progressed.	3 (7.3%)	3 (7.3%)	2 (4.9%)	5 (12.2%)	8 (19.5%)	14 (34.1%)	6 (14.6%)
I felt eager to discover how the game continued.	2 (4.9%)	1 (2.4%)	5 (12.2%)	8 (19.5%)	9 (22%)	11 (26.8%)	5 (12.2%)
It was easy to know how to perform actions in the game.	1 (2.4%)	1 (2.4%)	3 (7.3%)	6 (14.6%)	4 (9.8%)	18 (43.9%)	8 (19.5%)
The actions to control the game were clear to me.	-	1 (2.4%)	4 (9.8%)	4 (9.8%)	9 (22%)	16 (39%)	7 (17.1%)

I thought the game was easy to control.	1 (2.4%)	2 (4.9%)	6 (14.6%)	2 (4.9%)	4 (9.8%)	13 (31.7)	13 (31.7)
The game was not too easy and not too hard to play.	-	1 (2.4%)	-	2 (4.9%)	10 (24.4%)	16 (39%)	12 (29.3%)
The game was challenging but not too challenging.	-	1 (2.4%)	1 (2.4%)	1 (2.4%)	4 (9.8%)	21 (51.2)	13 (31.7%)
The challenges in the game were at the right level of difficulty for me.	-	1 (2.4%)	-	4 (9.8%)	7 (17.1%)	14 (34.1%)	15 (36.6%)
The game informed me of my progress in the game.	-	-	1 (2.4%)	4 (9.8%)	15 (36.6%)	11 (26.8%)	10 (24.4%)
I could easily assess how I was performing in the game.	-	1 (2.4%)	1 (2.4%)	7 (17.1%)	16 (39%)	11 (26.8%)	5 (12.2%)
The game gave clear feedback on my progress towards the goals.	-	-	3 (7.3%)	11 (26.8%)	10 (24.4%)	12 (29.3%)	5 (12.2%)
I enjoyed the way the game was styled.	-	1 (2.4%)	3 (7.3%)	4 (9.8%)	4 (9.8%)	17 (41.5%)	12 (29.3%)
I liked the look and feel of the game.	-	1 (2.4%)	1 (2.4%)	1 (2.4%)	7 (17.1%)	19 (46.3%)	12 (29.3%)
I appreciated the aesthetics of the game.	-	-	1 (2.4%)	5 (12.2%)	5 (12.2%)	17 (41.5%)	13 (31.7%)
I grasped the overall goal of the game.	-	-	-	2 (4.9%)	5 (12.2%)	13 (31.7%)	21 (51.2%)
The goals of the game were clear to me.	-	-	1 (2.4%)	3 (7.3%)	4 (9.8%)	17 (41.5%)	16 (39%)
I understood the objectives of the game.	-	-	1 (2.4%)	3 (7.3%)	4 (9.8%)	17 (41.5%)	16 (39%)
I liked playing the game	1 (2.4%)	1 (2.4%)	-	3 (7.3%)	6 (14.6%)	17 (41.5%)	13 (31.7%)
The game was entertaining	1 (2.4%)	1 (2.4%)	1 (2.4%)	3 (7.3%)	9 (22%)	19 (46.3%)	7 (17.1%)
I had a good time playing this game	1 (2.4%)	1 (2.4%)	1 (2.4%)	4 (9.8%)	8 (19.5%)	16 (39%)	10 (24.4%)
